# Sustentacular screw placement with guidance during ORIF of calcaneal fracture: an anatomical specimen study

**DOI:** 10.1186/s13018-017-0580-5

**Published:** 2017-05-30

**Authors:** Chen Wang, Dichao Huang, Xin Ma, Xu Wang, Jiazhang Huang, Chao Zhang, Li Chen, Xiang Geng

**Affiliations:** Department of Orthopedics, Huashan Hospital, Fudan University, No.12, Middle Wulumuqi Road, Jingan District, Shanghai, China

**Keywords:** Calcaneal fracture, Assistant of guidance, Sustentacular screw, ORIF, Accuracy

## Abstract

**Background:**

The sustentacular screw is essential to maintain the stability of the subtalar joint during ORIF (open reduction with internal fixation) of calcaneal fractures. Currently, the screw is still inserted based on surgeons’ anatomical experiences and nearly 40% of screws are misplaced from the sustentaculum. Previous studies demonstrated some methods of sustentacular screw placement through anatomical measurements or navigation system. The purposes of this study are to design an assistant guidance device that can effectively improve the accuracy of sustentacular screw placement and to compare the accuracy of this technique with traditional screw placement based on experience.

**Methods:**

A customized guidance device is designed, aiming to improve the accuracy of sustentacular screw placement. Twenty cadaveric specimens are used in the present study. Ten specimens are allocated into the guidance-assisted group, and others are included in the traditional screw insertion group. A total of 40 sustentacular screw placements are performed in each group. Fluoroscopic images are obtained after each screw placement. Only the screw that captures the sustentaculum both on the lateral and axial X-ray views was regarded as an accurate placement.

**Results:**

The accuracy rate in the guidance-assisted group is 87.5% (35 out of 40 times of insertions) while in the traditional screw insertion group, the accuracy rate is 65% (26 out of 40 times of insertions). A significant difference is found between the two groups (*p* = 0.018).

**Conclusions:**

The guidance-assisted technique is a convenient approach that can effectively improve the accuracy of sustentacular screw placement during the ORIF of calcaneal fractures. This study provides a novel technique that significantly facilitates sustentacular screw insertion and improves its accuracy.

## Background

Calcaneal fractures comprise approximately 60% of tarsal bone fractures and approximately 2% of all fractures [1, 2]. These fractures can be intra-articular or extra-articular; intra-articular fractures comprise approximately 75% of all calcaneus fractures. The primary fracture line is oriented from plantar-medial to dorso-lateral and therefore creates the sustentacular fragment that varies in size based on the fracture line [1, 3–5].

The current surgery options for calcaneal fractures are closed reduction, percutaneous fixation, or open reduction with internal fixation (ORIF) [4]. The best method in achieving calcaneal anatomic reduction and morphology restoration of articular surface is believed to be ORIF [6].

Correct placement of the sustentacular screw is one of the critical procedures during the ORIF of calcaneal fractures [7, 8]. The functions of the screw have been demonstrated both biomechanically and clinically. Pang QJ [8] proved that sustentacular screw placement was essential for the stability of the posterior facet based on the finite element model. A clinical study [6] showed that the absence of a sustentacular screw in calcaneal fractures would cause the decrease of Bohler’s angle in long-term follow-up.

However, because of the lateral position during operation and the limited surgical exposure, accurate insertion of the sustentacular screw is technically difficult. Misplacement of the sustentacular screw jeopardizes many important structures around the sustentaculum, such as the subtalar joint, flexor digitorum longus, flexor hallucis longus, and neurovascular bundle, and may result in poor outcomes [6, 7, 9].

Previous studies have proposed some techniques to improve the success rate of sustentacular screw insertion. Bussewitz BW [9] determined the starting point and the inclined angle of the sustentacular screw in their anatomical study. He noted the screw should be inserted approximately 16 mm posterior to the ST reference point with an angle of approximately 30 ° from posterior and lateral to anterior and medial. However, this approach still greatly depends on the experience of the surgeons. Recently, the navigation system and the 3D fluoroscopic technique have been introduced to facilitate the screw placement; however, these techniques are relatively difficult to popularize because of the equipment limitations [10].

Therefore, the goals of this study are (1) to design an assistant guidance device that can be easily operated and can effectively improve the accuracy of sustentacular screw placement and (2) to compare the accuracy of guidance-assisted technique with traditional screw placement performed by an experienced foot and ankle surgeon. Our hypothesis is that with the help of the guidance device, the success rate of sustentacular screw placements will be improved.

## Methods

### Structure of guidance device

**Fig. 1:**
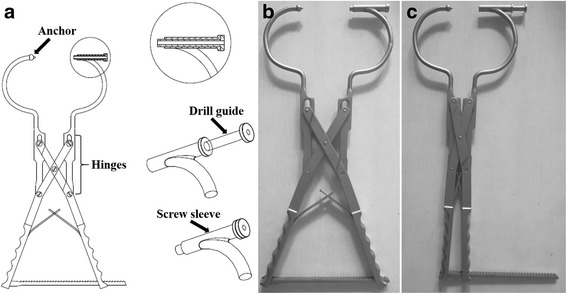
Customized guidance for sustentacular screw insertion. It consists of several essential parts (**a**) the anchor, three groups of hinges, screw sleeve, drill guide, and two clamp arms. The two arms of the clamp are combined through three groups of hinges. The drill guide is removable while the screw sleeve is fixed. The entry and exit of the screw sleeve are always in line with the anchor during opening (**b**) and closing (**c**) of the device

The appearance of the customized guidance device is shown in Fig. 1a. It consists of several essential parts: the anchor, three groups of hinges, screw sleeve, drill guide, and two clamp arms. The clamp arms are connected by the three groups of hinges. During the opening and closing process of the device, the entry and exit of the screw sleeve are always in line with the anchor on the opposite arm (Fig. 1b, c). The drill guide is removable and its center hole is 2.8 mm, which fits the 2.7 mm drill. The hole of the screw sleeve is 4.5 mm in diameter and is used to guide sustentacular screw insertion (Fig. 1a).

### Cadaveric specimen preparation

Twenty-five frozen below-the-knee specimens were recruited in the study. All the specimens were obtained from the voluntary body donation centre of ***Medical School, ***University. These specimens were observed to have no calcaneal deformity on anterior-posterior, lateral, and axial X-ray films by two experienced foot and ankle surgeons. The exclusion criteria were: (1) obvious deformity of ankle joint or mid-hindfoot in appearance; (2) obvious deformity, degeneration of mid-hindfoot, and severe osteoporosis showed on anterior-posterior, lateral and axial X-ray films. Two specimens with obvious degeneration of hindfoot joints and three specimens with severe osteoporosis were excluded. Finally, 20 frozen cadaveric specimens (14 right and 6 left) were included in the study. The specimens were preserved at −18 °C and were thawed for 12 h at room temperature before the experiments.

**Fig. 2:**
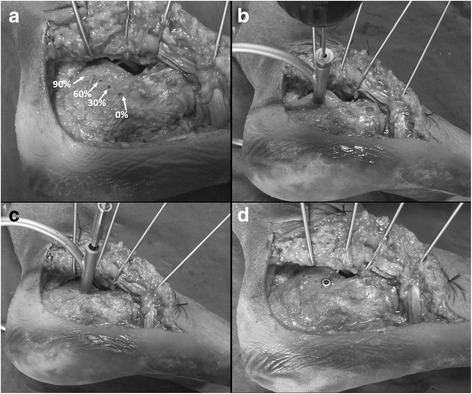
**a** Exposure of calcaneal lateral wall and positions of the four insertion start points. **b** The drill bit created a canal through the hole of inner sleeve. **c**–**d** Sustentacular screw was inserted through the outer sleeve

All of the specimens were placed in a lateral position and the extended “L-shaped” approach was performed to expose the lateral wall of the calcaneus and the posterior facet of the subtalar joint. Based on the research of Phisitkul P [11], the best screw entry level is located 15 mm below the posterior facet of the subtalar joint. Therefore, in the present study, four insertion entry points were marked on each specimen at this entry level, dividing the posterior facet into 0, 30, 60, and 90% as shown in Fig. 2a.

### Screw insertion with the guidance device

Ten specimens were selected for the guidance assistant group (group A). For each specimen, four sustentacular screw insertions were conducted with respect to each of the four entry points. All of the operations were completed by one orthopedic resident who has 3 years of orthopedic surgical experience. The sustentacular tali is projected at 1 cm below the anterior colliculi of the medial malleolar [7] and can be directly palpated. The anchor of the guidance was fixed at this projected point while the screw sleeve was located at each of the four entry points on the calcaneal lateral wall (Fig. 2b). A 2.7-mm drill bit was inserted through the drill guide and created the screw canal in the calcaneus (Fig. 2c). A 3.5-mm cortical screw was then inserted through the screw sleeve after removal of the drill guide (Fig. 2d).

### Traditional screw placement

The remaining ten specimens were used for traditional screw placements (group B). All 40 screw insertions were performed by one experienced foot and ankle surgeon (who had more than 10 years of orthopedic surgical experience and who had operated on 400 ORIF of calcaneal fractures) followed the methodology described by Bussewitz BW [9]. The surgeon was blinded to the study design before accomplishing all of the screw placements.

### Fluoroscopic images

**Fig. 3:**
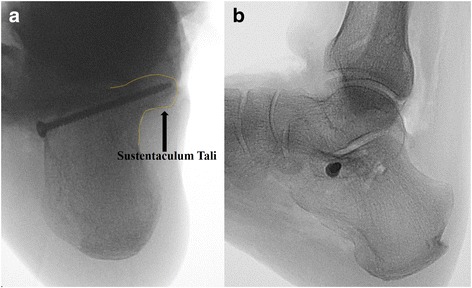
Fluoroscopic images showed that the screw engaged into the sustentaculum both in the axial (﻿**a**) and lateral (**b**) views

Both the lateral view and the axial view of the calcaneus were captured after each screw placement. The X-ray images were analyzed in blinded fashion by two orthopedic attendants. Only the screw that captured the sustentaculum both on the lateral and axial views was regarded as being accurately placed (Fig. 3). Different configurations were further evaluated when the screw missed the sustentacular tali: (1) screw inserted inferior to the sustentaculum; (2) screw inserted superior to the sustentaculum; (3) screw inserted anterior to the sustentaculum and; (4) screw inserted posterior to the sustentaculum. For each insertion, screw misplacement was recorded only in one plane that deviated more from the sustentaculum tali.

### Statistical analysis

SPSS 19.0 software was used to conduct statistical. The χ^2^ (chi-square test) was applied to compare the accuracy rate between the two groups. The statistically significant level was set as *p* < 0.05.

## Results

**Fig. 4:**
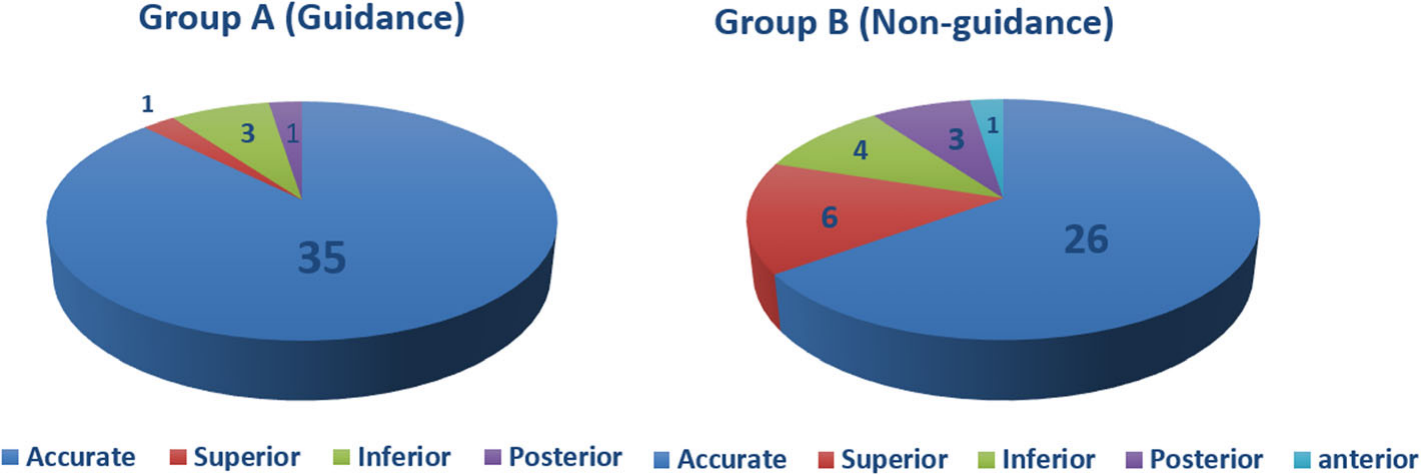
Numbers of accurate placements and misplacements of the sustentacular screw in both groups

The screw placement results were shown in Table 1 and Fig. 4. With the guidance assistant, the screw accurately engaged into the sustentaculum in 35 placements (accuracy rate 87.5%). For the traditional screw insertion, 26 of 40 screw placements accurately captured the sustentaculum (accuracy rate 65%). A significant difference was found between the two groups (*p* = 0.018).

**Table 1:** Accuracy of sustentacular screw placement in both groups

Groups	Accurate placements	Misplacements	Accuracy(%)	*χ* ^2^ value	*p* value
Group A	35	5	87.5	5.59	0.018
Group B	26	14	65.0		
Sum	61	19	76.25

**Fig. 5:**
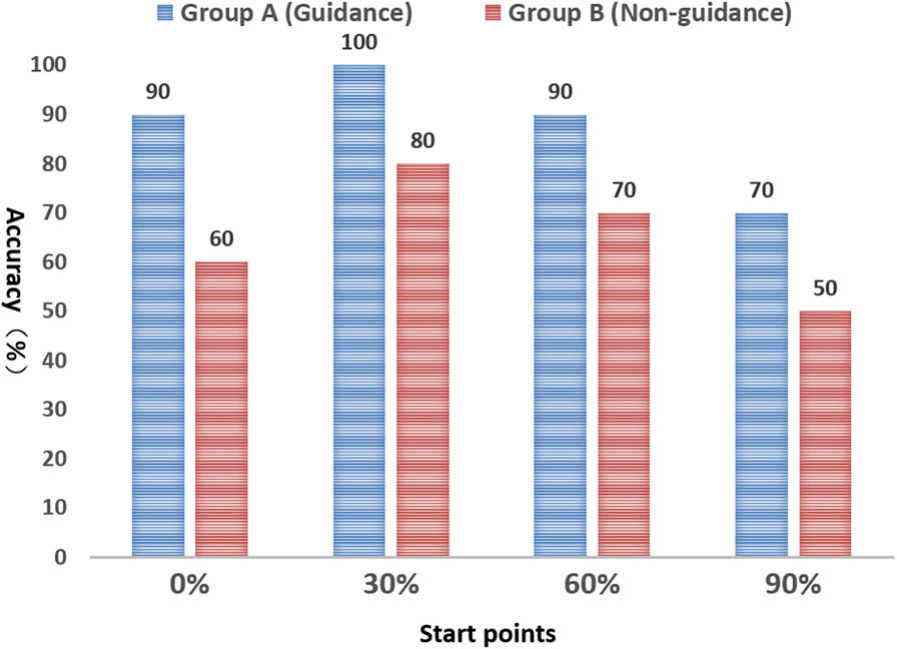
Success rate of sustentacular screw insertions at each insertion start point

For each of the four insertion start points, screw accuracy in both groups was shown in Fig. 5.

## Discussion

Calcaneal fractures are commonly seen injuries in the foot and ankle, comprise approximately 2% of all fractures and account for approximately 65% of all tarsal bone fractures. When the calcaneal fracture occurs, the calcaneus can be compressed and the posterior articular surface be comminuted because of the vertical pressure from the talus. However, as a result of the strong stabilized structures of the tendons and ligaments around the sustentaculum tali, the displacement of the sustentaculum tali rarely occurred in the calcaneal fractures [12]. Therefore, the sustentacular fragment is frequently the foundation for calcaneal fracture reduction because of its limited displacement [6, 7].

Although no consensus has been achieved regarding the treatment of calcaneal fractures, ORIF is still the primary method in most conditions [1, 4, 5]. Previous clinical and biomechanical research has demonstrated that anatomical reconstructions of displaced intra-articular calcaneal fractures contributed to providing better long-term outcomes compared with other therapies. Buckley R [13] conducted a multicenter RCT of 471 displaced intra-articular calcaneal fractures and found that ORIF reduced the risk of arthrodesis by 83% compared with non-operative treatment. Marcel Csizy [14] analyzed the clinical outcome of patients who failed after primary conservative or ORIF treatment of a displaced intra-articular calcaneal fracture. They discovered that the ORIF treatment had one-sixth the likelihood of secondary subtalar fusion.

Recent studies have also confirmed that sustentacular screw placement is essential to restore the stability of subtalar posterior facet [2, 8, 9]. Incorrect placement of the sustentacular screw usually injures surrounding important structures and might result in severe complications such as arthritis of the subtalar joint, persistent pain and swelling of the medial hindfoot, chronic impingement of the FHL tendon, paraesthesia of the medial planta pedis, and tarsal tunnel syndrome [15–17].

However, the success rate of accurate screw placement was not high enough when only relying on the anatomical experience of the surgeons [10, 18, 19]. Geerling J [10] found that during operations, sustentacular screws were intra-articular in 24% of the cases and penetrate the medial calcaneal cortex in 16% patients as documented by 3D scans.

Some previous studies contributed to improving the accuracy of screw placement. Phisitkul P [11] determined the best trajectory for sustentacular screw insertion based on computational 3D bone model research. They found that the sustentacular screw should be approximately 40 mm in length and should start 15 mm below the posterior facet of subtalar joints. Bradly W [9] described the anatomical morphology and anatomic landmarks of sustentaculum tali that can help target the “constant fragment” screw placement for calcaneal fractures. However, these studies were based anatomically on the structure and morphology of sustentaculum tali that still greatly rely on the surgeons’ experience.

A recent study reported using navigation techniques to reduce errors when placing sustentacular screws. Gras F [20] evaluated the accuracy of sustentacular screw placement using different navigation procedures including 2D navigation, 3D navigation, and fluoro-free navigation compared to the traditional procedure. Although they discovered that the 3D navigation procedure provides the best orientation, there was no significant improvement observed in the accuracy of screw placement compared with the traditional method. Furthermore, these techniques require complicated equipment and infrastructure in addition to experience in the surgical team.

In the current study, the success rate of traditional screw placement was 65% and was consistent with a previous study that reported 61% [10]. With the assistance of the guidance technique, significantly higher accuracy could be achieved even when undergoing the procedure by a less experienced surgeon.

However, we noticed that, in the guidance group, there were still 5 out of 40 screw placements in which the screws were misplaced primarily, these were inferior placements. We considered that these failures were caused by one specific defect of the current guidance design. The anchor was relatively short, and there was an enlarged ball structure behind the anchor. Therefore, the anchor could not firmly fix to the sustentaculum and tended to move inferiorly with the skin when tightening up the guidance.

The present study demonstrated that in the four insertion entry points, the 30% entry point was associated with the highest accuracy while the 90% entry point had the lowest successful rate in both the guidance-assisted group and the traditional group (Fig. 5).

There were several limitations in the present study. First, the sample size was relatively small in both tested groups. Even with 40 screw placements, the guidance-assisted approach showed its obvious advantages over the traditional experience-based approach. Second, some defects still existed in the current design of the customized guidance as explained above and may result in inferior misplacement of the sustentacular screw. However, after certain modifications, the success rate of sustentacular screw placement may be further improved. Third, this study was in vitro and has not been proven in clinical trials.

## Conclusions

The guidance-assisted technique is a convenient approach that can effectively improve the accuracy of sustentacular screw placement during ORIF of a calcaneal fracture. This technique may be promising in future clinical application.

## Abbreviations

ORIF: Open reduction and internal fixation
RCT: Randomized controlled trial
